# A late origin of the extant eukaryotic diversity: divergence time estimates using rare genomic changes

**DOI:** 10.1186/1745-6150-6-26

**Published:** 2011-05-19

**Authors:** Diana Chernikova, Sam Motamedi, Miklós Csürös, Eugene V Koonin, Igor B Rogozin

**Affiliations:** 1 National Center for Biotechnology Information, National Library of Medicine, National Institutes of Health, Bethesda, MD 20894, USA; 2 Department of Computer Science and Operations Research, Université de Montréal, Montréal, Québec, Canada

**Keywords:** bilateria, opisthokonts, angiosperms, last eukaryotic common ancestor, molecular dating

## Abstract

**Background:**

Accurate estimation of the divergence time of the extant eukaryotes is a fundamentally important but extremely difficult problem owing primarily to gross violations of the molecular clock at long evolutionary distances and the lack of appropriate calibration points close to the date of interest. These difficulties are intrinsic to the dating of ancient divergence events and are reflected in the large discrepancies between estimates obtained with different approaches. Estimates of the age of Last Eukaryotic Common Ancestor (LECA) vary approximately twofold, from ~1,100 million years ago (Mya) to ~2,300 Mya.

**Results:**

We applied the genome-wide analysis of rare genomic changes associated with conserved amino acids (RGC_CAs) and used several independent techniques to obtain date estimates for the divergence of the major lineages of eukaryotes with calibration intervals for insects, land plants and vertebrates. The results suggest an early divergence of monocot and dicot plants, approximately 340 Mya, raising the possibility of plant-insect coevolution. The divergence of bilaterian animal phyla is estimated at ~400-700 Mya, a range of dates that is consistent with cladogenesis immediately preceding the Cambrian explosion. The origin of opisthokonts (the supergroup of eukaryotes that includes metazoa and fungi) is estimated at ~700-1,000 Mya, and the age of LECA at ~1,000-1,300 Mya. We separately analyzed the red algal calibration interval which is based on single fossil. This analysis produced time estimates that were systematically older compared to the other estimates. Nevertheless, the majority of the estimates for the age of the LECA using the red algal data fell within the 1,200-1,400 Mya interval.

**Conclusion:**

The inference of a "young LECA" is compatible with the latest of previously estimated dates and has substantial biological implications. If these estimates are valid, the approximately 1 to 1.4 billion years of evolution of eukaryotes that is open to comparative-genomic study probably was preceded by hundreds of millions years of evolution that might have included extinct diversity inaccessible to comparative approaches.

**Reviewers:**

This article was reviewed by William Martin, Herve Philippe (nominated by I. King Jordan), and Romain Derelle.

## Background

Estimation of divergence dates for biological taxa from molecular data is a perilous exercise fraught by artifacts which become progressively more severe for events further in the past [1,2]. There are many factors that hamper molecular time estimates, especially for ancient events. Some of the most important problems are violations of the molecular clock, uncertainty in tree topology and branch length estimates, and the paucity and inaccuracy of fossil-based calibration points [2-4]. However, all these difficulties notwithstanding, knowing the dates of the major events in the evolution of life as precisely as possible is indispensable to connect biological evolution with the data of geochemistry and geology, and so to reconstruct the history of life on earth.

Arguably, dating is particularly important and interesting when it comes to the earliest divergence events in the evolution of eukaryotes. The deep phylogeny of eukaryotes is an extremely difficult and controversial problem. The concept of eukaryotic phylogeny that comes closest to being the current consensus maintains that there are 5 or, possibly, 6 distinct major branches, or supergroups, in the eukaryotic domain of cellular life, namely, unikonts (an assemblage that includes opishtokonts (metazoa, fungi, and related protists, and amoebozoa, with the latter considered a distinct supergroup in some studies), plantae, chromalveolata, excavates, and rhizaria [5-8]. Regardless of the exact status and composition of each individual supergroup, it appears that several major branches of eukaryotes diverged in a "Big Bang-type" event, whereby the internal branches in the tree are extremely short, so much so that the "true" tree topology might be undecipherable [9-11] given the intrinsic problems of deep phylogenetic reconstruction [2,12]. Although several attempts have been made to resolve the deepest eukaryotic branching by bringing some of the supergroups together into "megagroups" and rooting the tree [13-18], the relationships between the supergroups currently cannot be considered resolved, and the prospects for a conclusive solution are uncertain.

If the supergroups of eukaryotes indeed diverged in a rapid succession during an explosive phase of evolution, the problem of estimating the age of the Last Eukaryotic Common Ancestor (LECA) assumes a special importance. An accurate determination of this key date would be essential for tying the primary radiation of eukaryotes to specific events in the geological record and possibly identifying the factor(s) that triggered the rapid radiation.

The published time estimates for the divergence of eukaryotic taxa based on protein sequence analysis vary widely even for relatively recent evolutionary events due to the uncertainty of the fossil record, substantial rate variation and other problems of molecular dating [1,2,12,19,20]. Not unexpectedly, the uncertainty of the age estimates for LECA is much greater. The estimates vary approximately twofold if not more, from the most recent ones at ~1.1-1.2 billion years ago (Gya) [3,21,22]; to the most ancient ones at ~2.3-2.7 Gya [23,24]. Other studies have estimated the date of the divergence between phototrophic eukaryotic groups at ~1.6 Gya, with the implication that the primary radiation occurred before that date [25].

According to the fossil record, eukaryotes were already well diversified by ~ 1,500 Mya at the latest; however, these fossils cannot be clearly associated with any of the extant eukaryotic lineages [26-29]. In contrast to these findings of unclassifiable, even if apparently eukaryotic fossils, stands the single report the identification of a distinct multicellular eukaryotic fossil that appeared indistinguishable from the extant red algae of the genus *Bangia *and has been named *Bangiomorpha pubescens *n. gen., n. sp [30]. This fossil comes from the ca. 1.2 Gya Hunting Formation, so if this date is correct, the divergence of the eukaryotic supergroups and the differentiation of the major forms, including multicellular ones, within the supergroups obviously occurred earlier. Macroscopic fossils thought to represent primitive multicellular eukaryotes have been recently discovered in ~2.1 Gya shales [31] but these claims need to be taken with much caution considering the closely similar appearance of cyanobacterial mats [32].

An even older origin of eukaryotes has been inferred from the existence of apparent eukaryotic bio-markers such as C27-C29 steranes as far back as 2.7 Gya [26,33]. However, several lines of subsequent evidence seem to discredit these indications as artifacts [12,34,35].

We were interested in applying the genome-wide analysis of rare genomic changes associated with conserved amino acids (RGC_CAs) [36] to date the divergence of extant eukaryotes. Lately, the analysis of RGCs that can be exemplified by diagnostic gene fusions, domain architectures of proteins, or features of genome architecture such as insertions of mobile elements became an increasingly popular approach to study evolutionary relationships, given that these characters appear to be less prone to various artifacts than standard methods of molecular phylogeny [36-39]. Although it can be argued that RGC-based methods effectively employ parsimony and so would be subject to the same artifacts as maximum parsimony methods in sequenced-based phylogenetic analysis, this would not be the case if the RGCs were free of homoplasy (parallel changes and reversals) which is the primary problem for the maximum parsimony methods [36,40]. Conceivably, if the analyzed changes are indeed rare and their number is sufficiently large, the effect of homoplasy would be minimized [36,41]. It should be noticed that molecular phylogeny methods that employ sophisticated models of sequence evolution, usually within the maximum likelihood framework, are not without their own serious problems that are related, mostly, to model over-specification and mis-specification (proverbial attempts to "fit an elephant") [42-45]. Application of sequence-based phylogenetic methods within the phylogenomic approach has the potential to substantially increase the resolution power, but also poses challenges owing to different optimal models of evolution for different genes [46-49]. The pitfalls that are inherent in even the most advanced maximum likelihood and Bayesian methods, in particular, in the phylogenomic setting, stimulate the search for RGCs that are most suitable for phylogenetic analysis and molecular dating. In general, resolving complicated phylogenetic problems requires reconciliation of conventional phylogenomic approaches and RGC [12]. The RGC_CAs method combines some of the advantages of RGC and traditional phylogenetic analysis because the analyzed characters are both rare (amino acids that are conserved in multiple, distantly related species) and sufficiently numerous to allow robust statistical testing [36].

We applied the RGC_CA analysis to generate time estimates for some key events in the evolution of eukaryotes. The combined results of RGC_CA analyses using different methods point to a "young" LECA (~1.2 Gya) while yielding realistic and consistent time estimates for other major divergence events in the evolution of eukaryotes. Such a late date for the primary radiation of the extant eukaryotes suggests a long stem phase in the evolution of eukaryotes and has major biological implications.

## Results

### Approach and rationale

**Figure 1:**
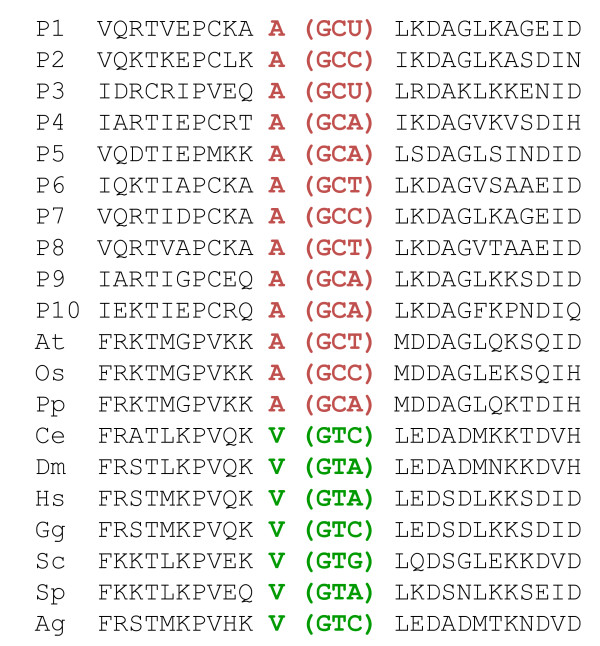
**An example of a RGC_CA used in this study**. The data are for KOG0100 (heat shock proteins). Using the notation described under Methods, P1 = ... = P10 = At = Os = Pp **≠ **Dm = Ag = Hs = Ce = Gg = Sc = Sp. The ancestral amino acid is shown in red, the opisthokont-specific substitution is shown in green. The corresponding codons extracted from the underlying nucleotide sequence alignments are shown in parentheses. Species abbreviations: *Homo sapiens *(Hs), *Caenorhabditis elegans *(Ce), *Drosophila melanogaster *(Dm), *Saccharomyces cerevisiae *(Sc), *Schizosaccharomyces pombe *(Sp), *Arabidopsis thaliana *(At), *Anopheles gambiae *(Ag), *Oryza sativa *(Os), *Physcomitrella patens *(Pp), *Gallus gallus *(Gg), and 10 outgroup prokaryotic species (P1...P10).

**Figure 2:**
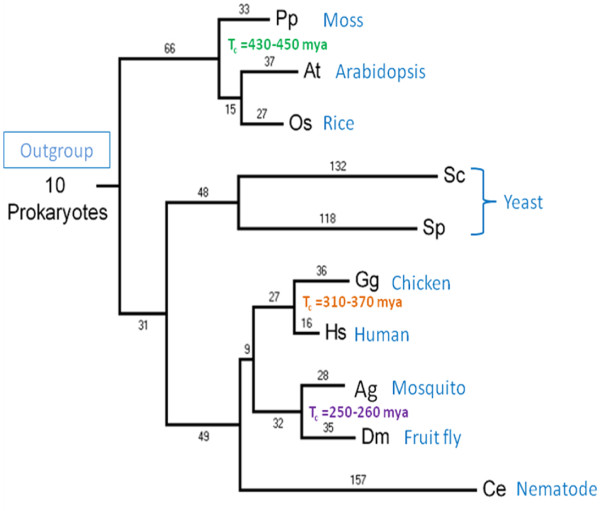
**The phylogeny of eukaryotes adopted in this study**. The figure shows the coelomate scenario (the ecdysozoa scenario is not shown). The numbers at the branches indicate the numbers of RGC_CAs which are used to measure the branch length. Tc, calibration time interval, Mya.

**Figure 3:**
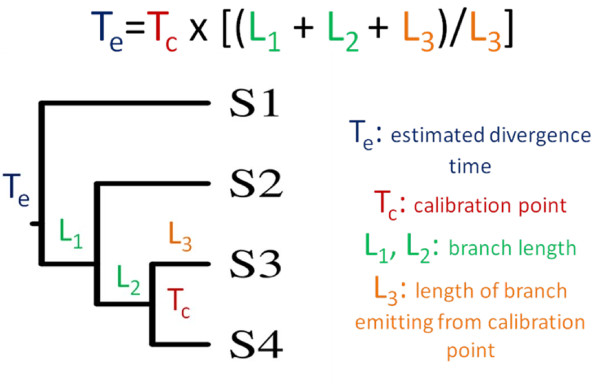
**Estimates for a divergence time T**_**e **_**corresponding to a calibration point T**_**c **_**and the branch path L**_**1 **_**+ L**_**2**_.

The RGC_CA approach [36,41] involved the analysis of amino acid residues that are conserved in most of the included eukaryotes, with the exception of a few species, and the prokaryotic outgroup. The assumption is that any character shared by the majority of eukaryotes and the 10 diverse prokaryotes (the outgroup) is the ancestral state, and accordingly, the species with a substitution in the given position possess a derived state (one, two or three nucleotide substitutions are allowed, Figure 1). Previous analyses have shown that amino acid changes that meet these criteria are rare and therefore the frequency of parallel emergence of such characters in different lineages is expected to be low [36,41]. This property is critically important for an RGC because homoplasy (parallel changes and reversals) is one of the primary causes of artifacts in phylogenetic reconstructions [36,40]. To assess the effect of homoplasy, we calculated the total number of RGC_CAs (1339 characters) and the number of RGC_CAs that are actually used for branch length estimates (1132 RGC_CAs) (Figure 2) (see Methods and Figure 3 for details). The difference between these two values is the number of RGC_CAs that are not compatible with the species tree (Figure 2) and so are most likely to result from homoplasy. Thus, the level of homoplasy (~15% of the RGC_CAs) was relatively low although some biases caused by homoplasy could not be ruled out.

The estimates of divergence times were obtained using the respective calibration times (point or range) and the branch lengths calculated from the RGC_CA analysis (see Methods and Figure 3 for details). After calibrating the RGC_CA approach using well established dates for the divergence of vertebrate taxa, we employed it in combination with four methods for molecular dating to obtain time estimates for four major divergence events: the primary radiation of the major eukaryotic lineages, i.e., the age of LECA; the radiation of opisthokonts; the radiation of animals; and divergence of monocot and dicot plants. Although the deep topology of the eukaryotic tree remains uncertain, we assumed that the plant-opisthokont divergence corresponded to the primary split; this is compatible with the results of independent attempts to root the eukaryotic tree. Accordingly, the time estimate of this divergence was taken as a proxy for the age of LECA. As discussed in the Introduction, there is a single dated red algal fossil that seems to point to an ancient origin of this group of eukaryotes. We certainly could not ignore this data point but felt that it had to be treated with special caution. So we first describe date estimates made without the red algal calibration point and then, in a special section, address the results obtained when this point is included. A detailed description of the results obtained with the RGC_CA approach is given in the Additional file 1.

### Calibration of the RGC_CA approach using vertebrate divergence dates

The concept of molecular clock-like behavior of phylogenetic characters is a controversial issue although these characters are frequently used for divergence time estimates (see Introduction). In a previous study, we for metazoans and demonstrated a reasonable consistency of the time estimates for the divergence of metazoan taxa obtained using RGC_CAMs [36]. The rates of RGC_CAM appearance have been shown to be, approximately, the same in the analyzed terminal and internal branches of three animal clades (nematodes, insects and deuterostomes), suggesting that RGC_CAMs behave as approximate molecular clock [36]. In the present work, we calibrated the RGC_CA method using the dates for vertebrate taxa divergence reported by Benton and Donoghue [50] and compared the results with those obtained using conventional sequence alignments. We analyzed the alignments which included the original data set of 716 KOGs [51] to which orthologs from 8 vertebrate species [*Mus musculus *(Mm), *Canis familiaris *(Cf), *Monodelphis domestica *(Md), *Ornithorhynchus anatinus *(Oa), *Gallus gallus *(Gg), *Anolis carolinensis *(Ac), *Xenopus tropicalis *(Xt), *Danio rerio *(Dr)] were added using the COGnitor procedure as described in Methods.

The phylogenetic tree of vertebrates with branch lengths measured in RGC_CAs and the appropriate calibration time intervals [50] is shown in the Additional file 2. To analyze the molecular clock properties of RGC_CAs, we plotted the rates of RGC_CAs accumulation (uncorrected for multiple changes) starting from all terminal branches against midpoints of calibration intervals [50] (Additional file 2). The plot is nearly linear with a Pearson correlation coefficient of 0.71. We also calculated the standard Dayhoff distances from the sum total of amino acid replacements in ungapped concatenated sequence alignments using the CODEML program [52] (Additional file 2). The resulting plot was less linear compared to the RGC_CA plot (Additional file 2), with the correlation coefficient of 0.65. The observed better performance of RGC_CAs compared to conventional distances, in terms of linear dependence on divergence time (Additional file 2), even for short phylogenetic distances (some internal branch lengths are effectively 0, see Additional file 2) suggests that the RGC_CAs represent a reasonable approximate molecular clock and have potential for molecular dating.

### The primary radiation of eukaryotes: the age of LECA

The time estimates for the primary eukaryotic radiation are shown in Table 1. The mean of the estimates from all methods puts the age of LECA at ~1,130 Mya; the distribution of all estimates is shown in the Additional file 3.

**Table 1:** Time estimates produced with different calibration intervals used in this study

Program/time estimates (Mya)	LECA	Opisthokonts	Bilaterian animal phyla	Dicot/monocot
*- PAML mcmctree*				

Mean estimate	1253/1036	1140/895	719/598	331/322

Median estimate	1252/1014	1140/874	719/581	332/321

Std. Deviation	3/101	3/77	24/67	3/3

*- Multidivtime*				

Mean estimate	1293/895	1138/774	689/538	284/290

Median estimate	1292/892	1138/770	686/536	284/291

Std. Deviation	5/19	2/12	51/36	5/6

*- R8S*				

Mean estimate	1271/1196	1135/1014	745/686	329/344

Median estimate	1271/1186	1135/1003	745/682	329/344

Std. Deviation	8/106	3/86	39/26	0/6

*- Maximum Parsimony*				
Mean estimate	1457/1315	1267/1073	768/635	406/373

Median estimate	1457/1162	1267/994	768/601	406/328

Std. Deviation	10/480	10/342	16/147	34/140

For each data point, the numerator shows the results obtained with the plant, insect and red algal calibration intervals, and the denominator shows the results obtained with the plant, insect and vertebrate calibration intervals.

Multidivtime [53] gave a rather narrow range of recent *T*_*e *_estimates for the divergence of plants and opisthokonts (871 - 923 Mya), with the 95% confidence interval from 719 Mya to 1,161 Mya (Additional file 1). The PAML mcmctree program [52] yielded a broader range of somewhat older dates (935 - 1,197 Mya), with the 95% confidence interval from 702 Mya to 1,808 Mya (Additional file 1). The *T*_*e *_estimates produced by r8s [54] pointed to yet slightly older dates in the range of 1,104-1,308 Mya (Additional file 1).

Maximum parsimony produced a relatively narrow range of the *T*_*e *_estimates consistent with the results obtained with other methods (1,021 - 1,384 Mya) when the chicken calibration point was not used for time estimates (Additional file 1). With chicken included, a substantially wider (and unreasonable) range of the *T*_*e *_estimates (1,470 - 3,053 Mya) was produced, probably because of the major branch length differences (e.g., 36 RGC_CAs on the chicken branch compared to 18 RGC_CAs on the human branch) and the broad date range (310-370 Mya) for that led to over-dispersion (Additional file 1).

**Figure 4:**
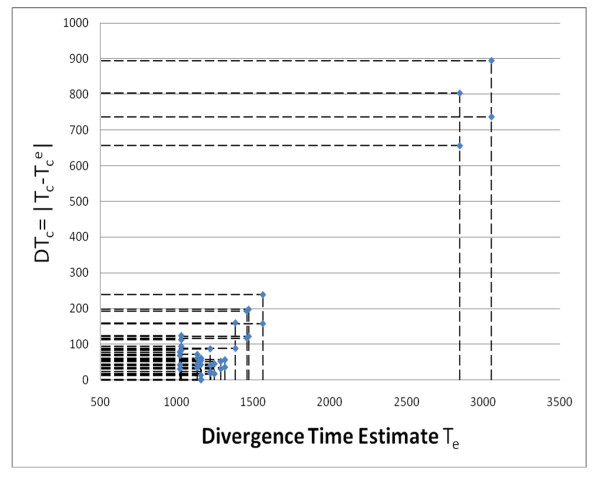
**Cross-validation: the dependence of *T*_*e *_on *DTc *= | *T*_*e *_*- T*^*c*^_*e *_|**. Estimates for the divergence times of extant groups of eukaryotes are plotted against the absolute value of the difference between the value of a calibration point X and an estimate for X. Results obtained for all three calibration intervals (Figure 2) were pooled together.

This problem prompted us to assess the reliability of the calibration points using cross-validation analysis [55]. The general approach used for cross-validation is to check the reliability of molecular clocks by comparing an actual calibration point and a parsimony-estimated divergence time for a specific divergence event (using other calibration points). Small differences between observed and estimated values suggest that the molecular clock assumption is (approximately) valid for a given dataset and tree topology, whereas large differences indicate that molecular clock is not applicable. The plot of *T*_*e *_*vs. *| *T*_*c *_*- T*_*e*_^*c *^|, i.e., estimates for the divergence times between extant eukaryotes versus the absolute value of the difference between a calibration time *X *and the estimate for the same divergence event, is shown in Figure 4. Clearly, the extremely large *T*_*e *_values as well as the smallest *T*_*e *_values correspond to poor estimates for the respective calibration points, i.e., large differences between the estimates and calibration times. Thus, the extremely high *T*_*e *_estimates obtained with the chicken calibration point indeed are most likely artifacts caused by the long branch leading to chicken.

In general, only a small fraction (4 out of 60) of the *T*_*e *_estimates for the primary radiation of eukaryotes are older than 1.6 Gya. The reasonable consistency of the obtained time estimates and the typically narrow range (with the exception of the outlier values given by the calibration interval for the human-chicken divergence discussed above; see Additional file 3) is compatible with the approximate molecular clock properties of the RGC_CAs (see above). Specifically, the rates of RGC_CAs appearance were, approximately, the same in the analyzed terminal and internal branches of the tree except for some extreme cases of rate variability. This conclusion is further supported by the observation that the paths from LECA to different terminal branches of the analyzed tree have similar lengths (Figure 2 and Additional file 4). For example, the lengths of the paths that lead from LECA to human (123 RGC_CAs) and to Arabidopsis (118 RGC_CAs) (Additional file 4) are statistically indistinguishable (P = 0.75). Long branches are characteristic of fast-evolving taxa such as unicellular fungi and nematodes (Additional file 4). Apart from these taxa, there were no substantial differences between the lengths of paths from LECA to plants and animals (Additional file 4) although the rate of evolution of plants was systematically somewhat lower compared to animals resulting in statistically significant differences for some of the comparisons (Additional file 4).

### Opisthokont radiation

Numerous molecular phylogenetic studies have convincingly shown that two major eukaryotic groups, Metazoa and Fungi, derive from a common ancestor and form a clade known as the opisthokonts [56-58]. The monophyly of the opisthokonts is also supported by several shared ultrastructural characteristics, such as the presence of a unicellular motile stage bearing a single posterior flagellum and flattened mitochondrial cristae [57,58].

Although different methods produced a wide range of estimates (761-2,336 Mya), in general, the results of molecular dating with RGC_CA suggest a relatively late divergence of animals and fungi: 39 out of 60 time estimates are less than 1,000 Mya (Additional file 1). The average *Te *estimates for PAML, mcmctree, Multidivtime, r8s and maximum parsimony were 774 Mya, 895 Mya, 1,014 Mya and 1,114 Mya, respectively (Table 1). The 95% confidence interval for Muldivetime was reasonably narrow, from 622 Mya to 981 Mya (Additional file 1). The PAML mcmctree 95% confidence interval was much wider, from 601 Mya to 1,559 Mya (Additional file 1). The mean of the estimates from all methods yielded a divergence time for opisthokonts at 949 Mya (Table 2), a value that is well compatible both with the age of LECA estimated here, ~1,130 Mya, and the rapid divergence of the major clades after the primary radiation of eukaryotes. Again, the only gross outliers appear in the parsimony analysis with the chicken calibration point, most likely, for the reasons outlined above.

**Table 2:** Mean and median time estimates for all methods used in this study

Time estimates (Mya)		LECA	Opisthokonts	Bilaterian animal phyla	Dicot/Monocot
Mean time estimate		1319/1111/1173	1170/939/1006	730/614/646	337/332/332

Median time estimate		1296/1157/1179	1492/948/1004	745/595/623	333/321/328

Standard dev of estimates		96/438/398	66/311/290	43/133/129	56/123/113

Range	Min.	1250/871/871	1133/761/761	644/473/473	278/279/278
	
	Max.	1466/3053/3053	1279/2336/2336	787/1203/1203	438/957/957

For the mean time estimate, each program was given equal weight. For each data point, the first number shows the results obtained with the plant, insect and red algal calibration intervals, the second numbers shows the results obtained with the plant, insect and vertebrate calibration intervals, and the third number shows the estimate for all calibration intervals pooled.

### The radiation of bilaterian animal phyla and Cambrian explosion

For the radiation of the bilaterian animal phyla, PAML mcmctree, multidivtime and r8s produced consistent time estimates in the range of 495 - 719 Mya (Additional files 1 and 3). Maximum parsimony yielded a very similar range of *T*_*e *_values for divergence of animals (473 - 691 Mya) when chicken calibration point was not used for time estimates (Additional file 1). As in other cases, a much wider range of *T*_*e *_values (501 - 1203 Mya) was obtained when the chicken calibration point was included, probably because of artifacts caused by large branch length differences (see above).

**Figure 5:**
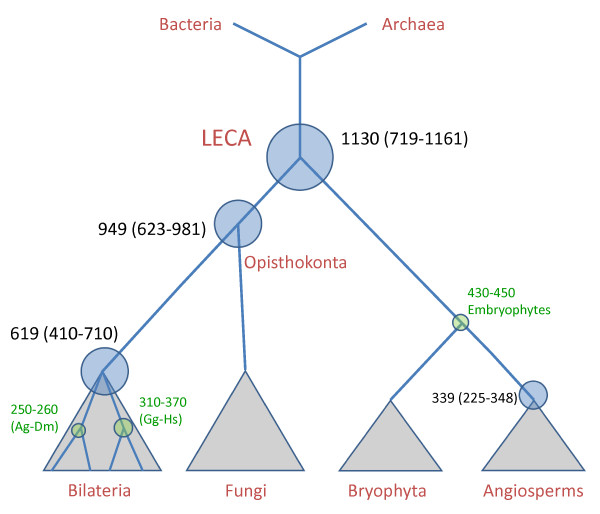
**The time scale for the evolution of eukaryotes suggested by the RGC_CA-based estimates obtained without the red algal calibration intervals**. Calibration intervals (green), mean time estimates for all programs (from Table 2) and Multidivtime 95% confidence intervals (in parentheses) are shown next to the respective nodes. The size of the circles is approximately proportional to the calibration intervals or 95% confidence intervals.

In general, these time estimates are much more recent than the previously reported early divergence dates for animal phyla, i.e., 970-1,040 Mya [59,60]. The mean RGC_CA-based estimate for the nematodes-insects-vertebrates divergence time is 619 Mya (Table 2 and Figure 5). These time estimates for the radiation of the animal phyla are better compatible with the Cambrian explosion, the well-known phenomenon of the rapid, almost simultaneous (on the geological scale) appearance of the animal phyla in the fossil record during the Cambrian[61,62], than the previously reported older estimates from molecular data [63]. Paleontological evidence suggests rapid, compressed cladogenesis soon after the origin of Metazoa approximately 600 Mya, with poriferans [64], cnidarians [65], and many of the bilaterian phyla making their appearance within 100 Mya [66]. Thus, inferences from these two independent lines of evidence (molecules and fossils) seem to support the origin of the Metazoa in the immediate aftermath of the global Neoproterozoic glaciation [64].

### Divergence of monocotiledonous and dicotiledonous plants

The divergence time of the monocot and dicot plants is yet another controversial issue. Fossil evidence suggests that flowering plants (angiosperms) first appeared ~140 million years (Mya) ago in the early Cretaceous [67]. The time estimates for the monocot-dicot divergence based on molecular data are highly variable but generally predate the angiosperm fossil record, ranging from 140-190 Mya [68,69] to ~200 Mya [70,71] or even 300-320 Mya [72-74]. Our time estimates (Figure 5) are consistent with those reported by Martin et al. and Brandl et al. [72-74]: all methods employed in this study produced a narrow range of the *T*_*e *_values for the divergence of monocot-dicot plants (306 - 433 Mya) except when the 370 Mya human-chicken calibration point was used for time estimates using maximum parsimony (Additional file 1). With this calibration point, substantial over-dispersion was observed for all analyzed divergence time estimates, so these results are likely to be a branch-length-related artifact (see above).

### Time estimates using the red algal data

The interval of dates for the fossil of a red alga that has been classified within a specific group of the extant red algae, the bangiophytes (1,198 ± 24) [30] certainly is one of the most important and controversial dates relevant for the temporal mapping the evolution of eukaryotes. Indeed, this appears to be the only report of a eukaryotic fossil from the early Proterosoic that belongs to an extant taxon and as such implies an old LECA. Of course, it has been reasonably argued that single fossils bear their own load of uncertainty in taxonomy identification and age determination [75]. Cumulative geological studies of the last decade seem to suggest that the safest age estimate for the Hunting Formation that contained the *Bangiomorpha *fossil is 1,100-1,222 Mya (Linda Kah, personal communication 2010). Accordingly, in the present study, we used this date interval for calibration after incorporating the orthologs from the red alga *Cyanidioschyzon merolae *into the analyzed alignments.

**Figure 6:**
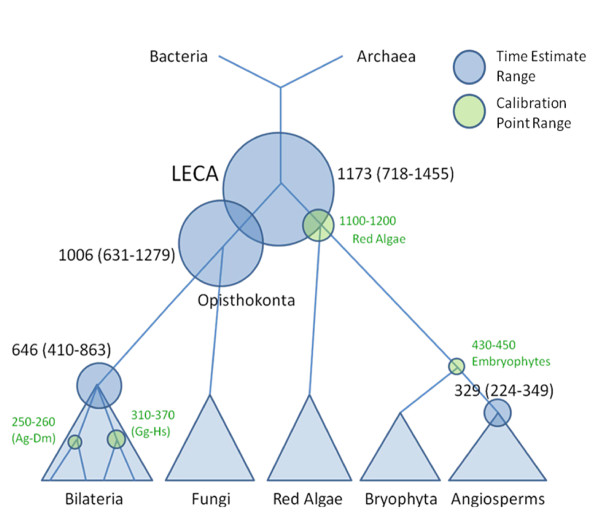
**The time scale for the evolution of eukaryotes from the RGC_CA-based estimates**. Calibration intervals (green), mean time estimates for all programs (from Table 2) and Multidivtime 95% confidence intervals (in parentheses) are shown next to the respective nodes. The size of the circles is approximately proportional to the calibration intervals or 95% confidence intervals.

The results obtained with and without using the red algal calibration interval are summarized in Tables 1 and 2. Predictably, these time estimates were systematically older compared to the estimates without the red alga (Tables 1 and 2) given that the length of the branch red algae to LECA is not negligible (Additional file 5). The distribution of time estimates for LECA was bimodal, with the substantial majority of estimated dates located in the 1250 - 1300 Mya interval and a minority of estimates around 1450 Mya (Additional file 6). This bimodality is likely to be an artifact of the maximum parsimony estimation which is based on the upper bound of the calibration interval (all estimates are in the range of 1448-1466 Mya) given that all other approaches yielded more recent dates (Additional file 1). The same bimodal shape is seen for the distribution of opisthokont time estimates (Additional file 6). Time estimates for bilaterian animal divergence were in the range of 710 - 790 Mya (Additional file 6) which is considerably older compared to the results obtained without the red algae (Table 1). A broad range of estimates was also obtained for the divergence of monocot and dicot plants (Table 1 and Additional file 6). In general, although the estimated dates were systematically older than those without the red algal calibration interval, the resulting overall time scale of eukaryotic evolution (Figure 6) was not dramatically different from that obtained without the red algal data (Figure 5) and still compatible with a "(relatively) young LECA".

## Discussion

Accurate reconstruction of the branching order for the major eukaryotic lineages is an extremely challenging task given the low information content of alignments of highly diverged sequences and the compressed cladogenesis that seems to be characteristic of the primary radiation of eukaryotes [5-7]. Dating these ancient divergences using molecular clock methods is even more difficult [1,2,19,20]. First, estimates of divergence dates are only meaningful if the phylogeny they are based upon is correct. Second, molecular dating requires accurate substitution models for the genes under consideration over billion-year time-scales as well as models that account for substitution rate variation across the tree branches [1,2,19,20]. Finally, divergence time estimation obviously requires reliable calibration points (dates) that only can be extracted from the fossil record and are associated with errors of several types including the error inherent in the dating of the associated geological strata; a systematic bias due to the fact that the true divergence date must be older than the first appearance of the descendant taxa in the fossil record; and the error associated with the extrapolation far outside the range of the calibration points that is inevitable in the estimation of ancient divergence times [2,19].

The use of RGCs has the potential to alleviate at least one of the key sources of error in molecular dating, namely evolutionary rate variation between branches of the tree (violation of molecular clock). Indeed, here we found that the rate of RGC_CA accumulation showed a stronger linear dependence on calibration divergence times inferred from the fossil record than the overall rate of substitution accumulation, suggesting that the RGC_CAs approximate molecular clock better despite the smaller number of data points. Further, cross-validation analysis supports the approximate molecular clock behavior of RGC_CAs because the rates of RGC_CA accumulation are, approximately, the same in the analyzed terminal and internal branches of the tree except for some extreme cases of rate variability. This conclusion is further buttressed by the narrow distribution of the distances from LECA to extant species measured in RGC_CA units.

A specific advantage of the RGC_CA-based dating is that this approach is relatively robust to errors in the branching order. The overall tree topology is not critical: what matters is only the correct topology of four branches involved in a particular estimate (Figure 3). With respect to the latter requirement, to estimate the age of LECA, we assumed that the divergence of plants and animals/fungi is the most ancient event in the evolution of eukaryotes. This assumption is compatible with the results of the eukaryotic root inference by different methods as well as the detailed phylogenetic analyses that led to the proposals of megagroups [13,16-18].

Different methods used in this study generally produced highly consistent time estimates for the primary divergence of eukaryotes (age of LECA), divergence of the animal phyla, divergence of opisthokonts and the dicot-monocot divergence. The distributions of all estimates span rather narrow time intervals, with the sole exception of the estimates obtained with maximum parsimony and the human-chicken calibration point at 370 Mya (Additional file 3).

The means of the estimates from all employed methods are 339 Mya for the dicot/monocot split; 619 Mya for the radiation of the Bilaterian animal phyla; 949 Mya for the radiation of opisthokonts; 1130 Mya for LECA (Figure 5). The inclusion of the still controversial red algal calibration date [30] along with the corresponding sequence data predictably pushed all the dates back (Figure 6). Nevertheless, even with this ancient date included, we obtained indications of a "young" age of LECA, with the mean of 1173 Mya (the implication of this estimate is a rapid, explosive post-LECA divergence of the main eukaryotic lineages so that less than 100 million years separates LECA from the appearance of red algae).

The origin and radiation of the angiosperms is a well-known difficult problem that fascinated biologists since the days of Darwin who called it "an abominable mystery" [76]. The current consensus in the plant evolution community seems to be a relatively late crown angiosperm radiation, at 140-180 Mya [77]. Even these estimates predate the appearance of indisputable angiosperm fossils that date to ~120 Mya [78]. Recently, an uncorrelated relaxed-clock analysis yielded an older, late Triassic date of approximately 217 Mya [79]. This date is at the low boundary of the range see in the present work (Figure 5). However, earlier studies that employed phylogenies of individual, highly conserved genes and a careful interpolation from several calibration points gave an estimate of ~300 Mya for the dicot/monocot split, in a good agreement with our present estimates [72-74]. A recent detailed study has dated the origin of the Coleoptera (beetles), i.e., the radiation of the major lineages of the holometabolous insects, earlier than previously suspected, at ~285 Mya [80]. If the radiation of angiosperms indeed predated the insect radiation, as suggested by the comparison of the respective estimates, the attractive hypothesis of plant-insect coevolution and the dependence of insect diversification on herbivory, that has been dismissed owing to the assumed late date of angiosperm radiation [80], might become relevant again.

The radiation of animal phyla in relation to the Cambrian explosion is possibly an even more controversial matter that the radiation of angiosperms. The appearance of the bilaterian phyla, which constitutes the "explosion proper", has been dated with considerable precision to 542-520 Mya [81]. Several estimates using molecular clock point to substantially older radiation dates: the extensive variation notwithstanding, all these studies estimated the divergence time between protostomes and deuterostomes to be >700 Mya [75,82,83], leading to the idea of a long interval of "invisible" animal evolution before the Cambrian explosion. However, the use of Bayesian relaxed molecular clock approaches yielded younger date estimates of 582 +/- 112 Mya [84] or 642-761 Mya (mean 695) [22] which are compatible both with the estimate obtained here and with the fossil record. Similar estimates have been obtained in another study that employed molecular clock but used invertebrate rather than vertebrate calibration points [85]. These younger dates have been subsequently questioned on methodological grounds [59]. Nevertheless, a comprehensive analysis using a variety of molecular clock methods combined with Bayesian techniques yielded estimates for the protostome/deuterostome split in the range of 733-641 Mya [63] which, taking the confidence intervals into account, is compatible with our RGC_CA-based estimate (although the inclusion of the red algal data yields a wider range that at the outside is compatible with ancient divergence long preceding the explosion). These convergent younger dates for the radiation of the bilaterian phyla support the hypothesis that the bilaterian cladogenesis took place during the latest pre-Cambrian period, the Ediacaran (635-542 Mya), whereas skeletons that are best preserved as fossils evolved during the Cambrian, creating the appearance of explosion [63,86,87].

The case of the fungi/metazoan split tells a story similar to that of the bilaterian radiation. Molecular clock methods produce date estimates as ancient as 1,600 Mya [88] but the Bayesian relaxed molecular clock approach gives much younger dates of 872-1,127 Mya (mean 983 Mya) [22] which overlaps with our estimate.

The age of LECA arguably is the most consequential date that we estimated. Our results suggests that the primary radiation of eukaryotes occurred about 1.1-1.2 Gya, or around 1.4 Gya at the earliest (when the red algal fossil data are used for calibration), in agreement with the results previously obtained with relaxed Bayesian molecular clock [3,22] but clearly not with estimates obtained with simpler molecular clock models that point to an ancient radiation of eukaryotes at ~2,500 Mya [24]. Given the convergence of independent dating approaches on the "young LECA", buttressed by the agreement between these methods on other key dates such as the bilaterian radiation and the fungi/metazoa split, it seems that the possibility that the diversification of all extant eukaryotes occurred no earlier than ~1.4 Gya should be taken seriously. The implications of a young LECA are manifold and might substantially affect our understanding of the origin and early evolution of eukaryotes.

The late origin of the extant eukaryotic diversity implies a substantial time gap between LECA and the earliest occurrence of (apparent) eukaryotic fossils which are confidently dated to times in the early Proterozoic (>1,500 Mya) [12,26-28,31]. Given that collectively the evidence for the ancient appearance of eukaryotes seems solid, the several hundred years of eukaryotic evolution before LECA requires explanation. A simple, straightforward scenario has been put forward by Philippe and Adoutte [89] who proposed that the diversification of eukaryotes was intimately linked to the mitochondrial endosymbiosis and that the beginning oxygenenation of the oceans thought to have started ~1,000 Mya [90] was the principal trigger of the evolution of the aerobic eukaryotes [12]. This scenario implies that the earliest eukaryotes were amitochondrial organisms, sometimes denoted archezoa [91-93]. However, an alternative, potentially more plausible scenario should be considered in light of the arguments that the mitochondrial endosymbiosis probably was the cause of eukaryogenesis rather than a relatively late capture of an α-proteobacterium by an archezoan [94-97]. The conclusion on the young LECA adds credibility to the ideas that the original main function of mitochondria was distinct from aerobic respiration and could involve other forms of metabolic symbiosis between the (archaeal) host and an α-proteobacterium (these hypotheses are also best compatible with the latest geochemical data that date the beginning of ocean oxygenation much later during the Neoproterozoic, perhaps, at 700-800 Mya [98]). Probably, the most coherent scenario of this type is the hydrogen hypothesis of Martin and Müller according to which the selective advantage of the symbiosis consisted in the production of molecular hydrogen needed for the host metabolism by the endosymbiont [99].

It seems likely that during the pre-LECA stem phase of eukaryotic evolution, the diversification of the primitive eukaryotes was limited as suggested by the fossil evidence [28]. It remains unclear what factors would trigger the explosive radiation that, according to the current estimates, occurred some 1.1 Gya. Regardless of the exact scenario of the evolution of eukaryotes, young LECA implies that we know next to nothing about a long and formative early part of the history of eukaryotes. Even if the early diversity of eukaryotes is incomparable to that created by the post-LECA radiation, there certainly were multiple lineages, and LECA obviously belonged only to one of these, and we know nothing about the rest. Indeed, inference of events that occurred during these "dark ages" is a formidable task because comparative genomics of eukaryotes cannot directly look past LECA. However, there are still ways to decipher some aspects of that early evolution, in particular, through detailed study of the numerous eukaryote-specific gene duplications [100] that, under the young LECA scenario, could have accumulated gradually over an extended period of time.

## Conclusion

Molecular dating is a formidably difficult enterprise due to multiple sources of intrinsic artifacts, yet there is no alternative to it for associating events in the evolution of life with the geological and geochemical history. Congruence between independent methods can greatly increase the confidence in the inferred dates. We show here that the RGC_CA behave like an approximate molecular clock and, when different estimation methods are used, consistently yield similar time estimates for key divergence events. In particular, these estimates point to a relatively late divergence of the major bilaterian lineages closely predating the Cambrian explosion and to a (relatively) young LECA, with the primary radiation of eukaryotes occurring between 1.0-1.4 Gya. The young LECA scenario implies several hundred million years of "hidden" evolution of eukaryotes for which virtually no data are available. If this is a valid depiction of the evolutionary history of eukaryotes, developing approaches to the study of the pre-LECA stem phase of eukaryotic evolution is a major challenge for evolutionary biologists.

## Methods

### Amino acid sequence alignments

Each of the 716 protein alignments (488,157 sites altogether) constructed from a previously delineated set of highly conserved clusters of eukaryotic orthologous genes, or KOGs [101] analyzed here included orthologs from 7 eukaryotic species with completely sequenced genomes: *Homo sapiens *(Hs), *Caenorhabditis elegans *(Ce), *Drosophila melanogaster *(Dm), *Saccharomyces cerevisiae *(Sc), *Schizosaccharomyces pombe *(Sp), *Arabidopsis thaliana *(At), and *Anopheles gambiae *(Ag) [51]. To these KOGs, probable orthologs from 66 prokaryotic genomes from the COG database [102] were added using a modification of the COGNITOR method [103]. Briefly, all protein sequences from the prokaryotic genomes are compared to the protein sequences previously included in the KOGs; a protein is assigned to a KOG when two genome specific best hits to members of the given KOG are detected. We added 10 prokaryotic orthologs (denoted below P1, ..., P10) to each KOG and required these prokaryotic orthologs to belong to 5 or more major prokaryotic clades (see Additional file 7) [104]. The requirement for the availability of 10 diverse prokaryotic orthologs was satisfied for 330 of the initially selected 716 KOGs. To the resulting mixed C/KOGs, probable orthologs from four other eukaryotic genomes, namely, those of rice *Oryza sativa *(Os), moss *Physcomitrella patens *(Pp), chicken *Gallus gallus *(Gg), and red algae *Cyanidioschyzon merolae *(Cm) were added using COGNITOR. For calibration of the RGC_CA approach, we analyzed the multiple alignments for the original data set of 716 KOGs [51] to which orthologs from 8 vertebrate species [*Mus musculus *(Mm), *Canis familiaris *(Cf), *Monodelphis domestica *(Md), *Ornithorhynchus anatinus *(Oa), *Gallus gallus *(Gg), *Anolis carolinensis *(Ac), *Xenopus tropicalis *(Xt), *Danio rerio *(Dr)] were added using the COGnitor procedure [103]. To minimize misalignment problems, only conserved, unambiguously aligned regions of the alignments were subject to further analysis. Specifically, we only analyzed positions surrounded by segments of protein alignments containing no insertions or deletions within a 5-amino acid window from each side.

### Rare genomic changes, RGC_CAs

To use the RGC_CA approach for the purpose of divergence time estimation [36,41], we analyzed amino acid residues that are conserved in most of the included eukaryotes, with the exception of a few species, and the prokaryotic outgroup. The assumption is that any character shared by the included 10 diverse prokaryotic outgroup species (P1, ..., P10) and the majority of eukaryotes is the ancestral state, whereas the deviating species possess a derived state (Figure 1). To simplify further presentation, we use the following notation: S1 ≠ S2 = S3 means that, for a conserved amino acid position in an alignment, species S2 and S3 share the same amino acid that is different from the amino acid in the species S1. Under this notation, for example, a human-specific RGC_CA is denoted by Hs ≠ At = Os = Sc = Sp = Dm = Ag = Ce = Pp = Gg = P1 = P2 = P3 = P4 = P5 = P6 = P7 = P8 = P9 = P10, whereas an RGC_CA shared by the fungi and animals is denoted by Sc = Sp = Hs = Dm = Ag = Ce = Pp = Gg ≠ At = Os = P1 = ... = P10.

Three sets of RGC_CA alignments were used for molecular dating using various approaches (see below), one consisting of 19 species (chicken and red algae not included) and 1339 amino acids positions, and two other alignments consisting of 20 species: 1) chicken included, red algae not included, 1161 amino acid positions; 2) chicken not included, red algae included, 1295 amino acid positions. Amino acid sequence alignments are available at the authors' Web site at ftp://ftp.ncbi.nlm.nih.gov/pub/koonin/RGC_CA/.

### Tree topologies and calibration intervals

For the alignment with 19 species, we used two calibration points or ranges (the maximum parsimony methods uses points whereas the other three methods we employed use took ranges): 260 Mya (250-260 Mya) for drosophila-mosquito divergence time [105,106] and 450 Mya (430-450 Mya) [107,108] for moss-angiosperm divergence time. For the alignments with 20 sequences, we used the above two calibration points/ranges and in addition either the 370 Mya (310-370 Mya) divergence time for mammals-chicken [109] or the unpublished Pb-Pb date of the oldest red algal fossil, 1122 Mya (1100-1222) [30] (Linda Kah, personal communication, 2010). The latter range is considered to be the most reliable estimate of the divergence date between the red algae and other plants (Linda Kah, personal communication, 2010; see discussion below). To estimate divergence times, four methods were employed: PAML mcmctree [52], Multidivtime [53], R8S [54], and an ad hoc implementation of maximum parsimony [36].

### PAML mcmctree

PAML's mcmctree [52] is a Bayesian phylogenetics program used for estimating divergence times with multiple calibration points. This program implements the Markov Chain Monte Carlo method, takes a bifurcating tree and accepts an upper and a lower bound on calibration points. A relaxed molecular clock (independent rates) model was used for all analyses. Divergence times were estimated using both the Dayhoff and the Jones-Taylor-Thornton (JTT) amino acid replacement models.

### Multidivtime

Multidivtime [53] is used for Bayesian analysis of evolutionary rates and divergence times. It employs the multivariate normal distribution to approximate the posterior distribution of divergence times. The program accepts an upper and lower limits on calibration points. Divergence times were estimated using both the Dayhoff and the JTT amino acid replacement models.

### R8S

The R8S program [54] estimates absolute rates of molecular evolution and divergence times. One of the methods R8S utilizes is a semiparametric method that relaxes the stringency of the clock assumption using smoothing methods. The semiparametric approach combines a parametric model with a different substitution rate on every branch with a nonparametric roughness penalty which penalizes the model if rates change too quickly from branch to branch. R8S uniquely does not take sequences as input, but instead takes branch lengths and tree topology. In the settings, we chose the Penalized Likelihood (PL) semiparametric method that utilized the Truncated Newton (TN) algorithm, which according to the authors of the program as best to use with the Penalized Likelihood method [54]. We used a smoothing value of 1 because we assumed that our model was not clock-like. Through the cross-validation procedure we found that a smoothing value of 1 was usually optimal. As the R8S program accepts only branch lengths and tree topology as input, we utilized branch lengths calculated by the maximum parsimony method described below.

### Maximum Parsimony

First, we estimated the branch length for each analyzed taxon in RGC_CA units [36]. For each species or group of species, we calculated the number of amino acid residues that are different from all other species (e.g., Sc = Sp **≠ **At = Os = Pp = Dm = Ag = Hs = Ce = Gg = P1 = ... = P10 for fungi). An example of eukaryotic phylogeny adopted in this study with calculated branch lengths is shown in Figure 2. To calculate divergence times, we assumed a strict molecular clock, where branch lengths corresponded to the number of molecular changes, and thus a fixed amount of time. Then, by using one calibration point at a time, and one branch path emanating from the chosen calibration point, we obtained the divergence time estimates. This method results in a number of estimates for any given divergence time, each corresponding to a different calibration point and branch path (Figure 3). The formula for the divergence time estimation for the path in Figure 3 is:

*T*_*e *_corresponds to estimated divergence time, *T*_*c *_is the calibration point, and *L*_*n *_corresponds to the length of branch *n*. In this formula, L_3 _is the length of the branch emitting from the calibration point (Figure 3).

## Competing interests

The authors declare that they have no competing interests.

## Authors' contributions

IBR and EVK incepted the study. IBR, DC, SM and MC implemented the tests and performed data analysis. IBR and EVK wrote the manuscript which was read, edited, and approved by all authors.

## Reviewers' comments

### Reviewer 1: William Martin, University of Duesseldorf

This is an interesting and worthwhile paper reporting calculations aimed to date major events in eukaryote evolution using a molecular clock approach to rare genomic changes. The authors use a multiplicity of methods, and get a range of values, which they openly report. Any criticism that could be leveled at molecular clocks of substitutions could be applied here. The authors recognize that, hence there is no need to get into the technical aspects of molecular dating approaches.

I have only a few points to discuss.

1. First, Bangiomorpha is probably the best early calibration point there is, and the authors even report here the unpublished Pb-Pb age of the fossil. Yes it is a single fossil, but it is probably the best early plant (red alga), and the authors basically throw it out, reporting estimates for major events in the abstract from analyses that exclude Bangiomorpha. In my opinion, this is a mistake, and it is the same mistake that Philippe made in ref 22, and that is why the present sets of estimates are so similar to those in ref 22. At the end of results we see much more reasonable ages for LECA, more compatible with the Javaux et al 1.45 Ga (billion years ago) material. The justification for excluding Bangiomorpha (a single find, yes, but well dates and exquisitely preserved) is not sound in my view. My inkling would be to report those dates using 1.2 Ga for plants in the abstract, too, and put suppl. information on the Bangiomorpha-based dates in the main text, not in the supplementary where no one will see it. As it stands, the best constrained early fossil is incompatible with dates put forth in this paper.

*Authors' response: We followed these suggestions by adding Table *2*and Figure *6* for the red algal calibration interval. We also mention the red algal calibration interval in the revised Abstract. Predictably, including this calibration point led to some revision of the time estimates but nevertheless, even with these data, LECA appears to be 1.4 billion years old at the most*.

2. I just outlined how Bangiomorpha was dismissed, and that that led to early dates. But, the dates are also too young to accommodate Tapannia and other finds from Javaux et al. dated at 1.45 (On the basis of better preserved material, Butterfield has reinterpreted Tappania from ref 26 as possibly being a fungus. Butterfield NJ (2005) Probable Proterozoic fungi. Paleobiology; v. 31; no. 1; p. 165-182). The criticism is severe, it is this: For Bangiomorpha, the authors opted to basically throw out the fossil because it does not fit their dates (when their dates actually should have been included that fossil in my view); for Javaux et al. ref 26, 1.45 Ga, the fossil material also does not fit their dates, but in THIS case the authors opt to interpret this as evidence for a long period of eukaryote evolution before the major modern groups appear. This is very inconsistent reasoning. Were they to be consistent, they would either have to a) throw out any unassignable acritarchs like those of ref 26 (too), just like they throw out Bangiomorpha or b) include Bangiomoprpha in which case 1.45 Ga material would no longer be outside the range. In the case of a) they loose their title and abstract evidence for extinct eukaryotic microbes (or "late" extant ones) because they would no longer accept the ref 26 or similar material as eukaryotes, putting them very close to Cavalier-Smith in terms of eukaryote age. In the case of b) everything is more or less normal, making this paper less of a splash perhaps but more consistent piece of work.

*Authors' response: As pointed out above, we have chosen option (b) by including Bangiomorpha, which has changed the estimated dates, but not that dramatically. The vast majority of these converged to the 1-1.4 Gya interval which is far from Cavalier-Smith's estimates. The title stands, accordingly*.

3. Supplementary table 6 says "with or without the red alga" but only one set of values is reported, presumably "without", because the ranges cannot possibly correspond to the LECA data in Suppl8. Or maybe I have missed something altogether here, which brings me to another point.

*This table was for all trees, with and without red alga. We replaced it with the table for the tree with red algal data. This table is consistent with the Additional file *6.

4. There are 10 supplemental files upon which the text and the arguments (hence the fabric of paper) rely. This annoyingly makes the referee and the reader click around through 12 windows to try to read the paper. I am tired of doing that anymore, and I think I am not alone. If I am not mistaken, J. Neurosci. has stopped accepting supplemental altogether, and that is a lead that one can consider. Biol. Direct is not printed, why not put all of the data that is essential to the paper in the paper and take out the stuff that is not essential? The axis labels in the Supplemental are problematic is several spots.

*Authors' response: We have merged three supplementary tables into the main Tables *1* and *2* and moved one supplementary Figure (now Figure *6) *to the main text. Still, several additional files remain. We believe that these files are indeed necessary but not as part of the main text which is already rather extensive and technical. Biology Direct has a convenient way to navigate through additional files in the HTML format*.

Points 1 and 2 seem severe in my view. My hunch is that many readers will see it similarly. Maybe I am wrong.

*Authors' response*: *As pointed out above, we took care of these points. The conclusions may have become less of a 'splash" but still stand*. *We added several qualifying remarks such as (relatively) young LECA etc.*

### Reviewer 2: Herve Philippe, University of Montreal (nominated by I. King Jordan)

I have three major problems with this manuscript that prevent me to perform a careful examination of the dating analysis per se.

#### 1) Problems with the dataset

Determining the orthology of genes is a very difficult issue (especially with species that underwent whole genome duplications, such as vertebrates). However, the methods used in this manuscript (716 KOGs from 7 eukaryotes plus a few additional taxa using COGNITOR-reference is missing-) are extremely rough and very likely produced numerous alignments containing non-orthologous copies. As an example, I analyzed the gene displayed on Figure 1. First, it turned out that the alignment is not from an RNA helicase but from carbamoylphosphate synthetase! Second, a phylogenetic analysis unambiguously demonstrated that sequences from plants are of cyanobacterial origin; in other words, the last shown RGC_CA is for a xenologous gene. It is therefore necessary to perform in depth controls to verify orthology.

Another important problem with the use of nucleotide sequences without quality controls is due to the existence of sequencing errors and incorrect intron/exon predictions. My own experience with these genomic data, obtained through the manual analysis of several hundreds of conserved genes, is that this represents a non-negligible problem, especially, in the case of RGC_CA: if the intron/exon prediction is incorrect for a single species at a highly conserved position, this will generate a RGC_CA. I think that the less accurate annotation of the Gallus versus the Homo genome provides a very likely explanation for the much longer branch of Gallus (36 versus 16 RGC_CA). Because the RGC_CA approach focuses on a very limited number of positions, it is highly sensitive to orthology and annotation errors.

Therefore, these two problems must be carefully assessed.

*Authors' response: We regret the problem with Figure *1. *The original version of Figure *1*was just an example of an RGC_CA. This particular RGC_CA came from our earlier studies but was never actually used for time estimates in the current work because, due to the relatively high variability in prokaryotes, this KOG did not pass the criteria applied for prokaryotic orthologs in the present study. We fully agree that it is only appropriate to use an example from this particular study, so in the revised version Figure shows the alignment for KOG0100 which is part of the present analysis.*

*Orthology identification certainly has its share of complications and pitfalls. We used a filtering procedure to delineate genes with putative cyanobacterial origin: KOGs with the best BLASTP hit to cyanobacteria were not used in this study (see Methods)*.

*We also compared different approaches for assigning orthology using manual checking and information on the location of introns. Our assessment indicates that the current version of COGnitor is reliable. All alignments with multiple RGC_CAs were manually checked, and several obvious cases of misidentification of orthology (e.g. complete loss of all catalytic residues characteristic of a given family of enzymes) were removed. Having said this, we generally attempted to minimize the extent of manual curation. The problem is that manual curation does not always guarantee 100% success because different researchers may have very different ideas of "undesirable" alignments. The subjectivity that is inherent in manual curation and elimination of "bad" alignments has the potential to severely bias phylogenetic analysis, so we attempted to limit the application of this approach*.

*In order to increase the robustness of the RGC_CA approach, we used reliable portions of amino acid alignments (no indels in region ± 5 aa around RGC_CAs). We also have experimented with amino acid conservation in the regions ± 5 aa; this does not change results, so the indel restriction appears to be rigorous enough*.

#### 2) Problems with the species sampling

I will not repeat all the comments made by Roy and Irimia (2008) and by referees of the Rogozin et al. article (Biology Direct 2008). But the arbitrary selection of a handful of eukaryotic and prokaryotic species when >100 and >1000 complete genomes are available is highly problematic. First, one should verify that the results are not sensitive to the choice of the species, because it has been abundantly demonstrated that evolutionary inference is sensitive to taxon sampling, especially when few species are used. Second, it is obvious that an extremely powerful method to minimize the effect of homoplasy is to use a large number of species. Let us assume that a position is evolving extremely rapidly (more than one change over each branch of the tree), but can only accept Ala and Thr for functional reasons. If one samples only 4 species, the probability to observe the same amino acid in all species is 1/8. So if one uses 488,157 sites as here, one would observe many "constant" positions that are in fact completely saturated. Obviously, if one uses 100 species, the probability of observing the same amino acid for all the species is almost 0 (1e-30). In contrast, if a position is really slowly evolving, the use of 4 or of 100 species will yield the same, correct, result. As a result, I don't see any reasons to use such a limited number of taxa: since the authors claim that RGC_CA is minimally affected by homoplasy, the results should remain identical.

*Authors' response: We experimented with different numbers of outgroup species by including 5 species (from at least three different lineages, this setting has been used previously for RGC_CAMs *[104]*), 10 species (from at least five different lineages, this setting also was has been previously for RGC_CAMs), 15 species (from at least five different lineages), and 20 outgroup (from at least five different lineages). The overall homoplasy level (fraction of RGC_CAs which are inconsistent with the species tree) was employed as the quality control criterion. We found that using 5 outgroup species produced a higher level of homoplasy (21%) compared to 10 species (15%), whereas further increase of the number of outgroup species did not substantially change the overall homoplasy level (13% for 15 species, 14% for 20 species)*.

*The number of eukaryotic species also might influence the homoplasy level of RGC_CAs. We analyzed eukaryotic species which we used in the previous paper *[36]*. We did not observe substantial differences in the sum of parallel changes and reversals for RGC_CAs when the number of species varied between 14 and 19 (for 14, 15, 16, 17, 18, and 19 species the fraction of parallel changes and reversals were 0.08, 0.09, 0.08, 0.0.7, 0.10, and 0.08, respectively).*

#### 3) Problems with RGC_CA

RGC_CAMs were defined as "amino acid replacements that require 2 or 3 nucleotide substitutions, in order to reduce homoplasy" (MBE 2007 24:1080, ref. 41). This was deemed necessary to address a relatively recent evolutionary history (Bilateria). I have to analyze

Figure 1 to discover that the authors relax the constraints of two or three nucleotide substitutions and now work on replacements that require only a single substitution. In other words, they accept more homoplasy to study a much more ancient phylogenetic question. I am unable to find the rationale behind that choice.

More importantly, I am not convinced by the reliability of the RGC_CA approach. As pointed out by Roy and Irimia, the fact that support for Coelomata is replaced by support for Ecdysozoa, when adding a close outgroup clearly demonstrates limitations of the RGC_CA approach. This is confirmed by the analysis of the position of Figure 1. I rapidly looked at this position in a few other species. In opisthokonts, which should have a highly conserved T, I observed that:

• Acyrthosiphon displays a S

• Ashbya displays a L

• Pyrenophora and Phaeosphaeria display a Q

• Podospora, Sordaria, Chaetomium and Neurospora display a A

• Cryptococcus and Trichosporon display a V

• there are 2 paralogs in Monosiga and they display A and T

In bikonts, which should have a highly conserved A:

• All kinetoplastids (~10 species) display a Q

• Apicomplexa display 5 S and 3 A

• Stramenopiles diplay 7 T, 2 A and 1 S

• Ricinus displays a T

In ~10 randomly chosen Archaea, which should have a highly conserved A:

• Natronomonas displays a T

• Sulfolobus diplays a S

• Pyrococcus diplays a V

In ~40 randomly chosen Bacteria, which should have a highly conserved A:

• Elusimicrobium, Lactobacillus, Clostridium, Catenibacterium, Ktedonobacter,

Sorangium and Waddlia display a T

• Dictyoglomus, Bachyspira, Escherichia and Methylobacillus

• Caulobacter display a G

• Ilyobacter and Bacteroides display a V

This quick analysis demonstrated that this position is not a RGC_CA, but had accepted many different substitutions. It is therefore very likely that this position will yield an unreliable signal when analyzed by maximum parsimony with a few species (i.e. an approach inefficient to detect multiple substitutions).

*Authors' response: There is no need to re-investigate a single RGC_CA in order to compare these characters with RGC_CAMs. We have previously reported such an analysis for numerous RGC_CAs *[36]. *Predictably, this analysis has shown that the homoplasy level of RGC_CAs is roughly 1.5-2 times higher compared to RGC_CAMs. In addition, below we present another RGC_CA vs. RGC_CAM comparison which also suggests that the homoplasy level is higher for RGC_CAs but not overwhelmingly so. However, there are two important reasons to use RGC_CAs instead of RGC_CAMs in this study. First, in this case, the dataset is much larger resulting in more reliable results and allowing one to use conventional phylogenetic methods. Second, the two Bayesian methods used in this study are not applicable to RGC_CAMs because of the requirement of at least two nucleotide substitutions for amino acid substitutions*.

These three major issues must be addressed before using these characters for molecular dating. I have nevertheless a few comments on the results. They are poorly presented, most of them being dispatched in several supplementary files, which make the evaluation difficult. I must also acknowledge that I was not able to fully understand the cross-validation (Figure 4), which is insufficiently explained.

*Authors' response*: *Additional explanations have been included on cross-validation in the main text and in the legend of Figure *4. *As pointed out above in the response to Reviewer 1, three supplementary tables have been merged into the main Tables *1* and *2, *and one supplementary Figure (now Figure *6) *has been moved to the main text. Still, several additional files remain. We believe that these files are indeed necessary but not as part of the main text. Biology Direct has a convenient way to navigate through additional files in the HTML format. As also noticed above, Biology Direct has a convenient way to navigate through the Additional Files in the HTML format (we do realize that it is less convenient during the review process and regret this inconvenience).*

Moreover, several statements are too vague (e.g. "demonstrated a reasonable consistency of the time estimates", "to be, approximately, the same in the analyzed terminal and internal branches"); the most important issue was the statement: "suggesting that RGC_CAMs behave as a relaxed molecular clock" (by the way, there is no comments in the text here on that fact that RGC_CAM could behave differently from RGC_CA, and the difference between the two approaches is not explained). Any set of homologous characters DO behave as a approximate molecular clock, even if it behaves as a strict molecular clock. The authors mistake "relaxed molecular clock" for "approximate molecular clock": approximate molecular clocks correspond to model rate variation over time (e.g. with autocorrelation).

*Authors' response: To the best of our understanding, the statements marked "vague" by the reviewer are sufficiently explained in the text, figures and tables. We agree with the reviewer that the properties of the RGC_CAs are appropriately described as those of "approximate molecular clock", so the necessary changes have been made throughout the manuscript. We regret this confusion which originated from the fact that the relaxed molecular clock assumption is involved in some of the methods used in this study*.

Finally, I have concern with this sentence: "Previous analyses have shown that amino acid changes that meet these criteria are rare and therefore the frequency of parallel emergence of such characters in different lineages is expected to be extremely low [36,41]." My understanding is that the authors consider that, because the number of positions displaying RGC_CA is very low, homoplasy is very rare at these positions. If my understanding is correct, there is a flaw in this logic. The only way to demonstrate that homoplasy is low at a given position is to analyze thousands of species for which phylogeny is approximately known and to verify that only a few substitutions are necessary to explain the data. On the other hand, one can look for positions that have a different amino acid in each of the 10 eukaryotic species under study and a same, but different, amino acid in each of the ten prokaryotic species. My prediction is that we will observe very few such positions (probably much less than RGC_CA positions), but this does not mean that these positions are homoplasy free.

*Authors' response: In the revision, "extremely" has been removed. As pointed out above, there is an inevitable trade-off between the number of characters and the level of homoplasy, so RGC_CAs are more homoplasy-prone than RGC_CAMs. An important question asked by the reviewer is how frequently RGC_CAs (which are 2-state characters) are in reality 3, 4, 5 etc.- states characters. We analyzed all columns located in the conserved portions of alignments and are conserved in the outgroup (similar to RGC_CA restrictions). We found for the RGC_CAM restriction that 93% of characters are truly 2-state RGC_CAM characters (6% and 1% of 3- and 4-state characters were found). The fraction of 3,4 et.-state RGC_CA-like characters is higher compared to RGC_CAMs (17%), however the fraction of 2-state RGC_CAs is still high (84%). The 3- and 4-state characters were relatively rare (13% and 3%). These results suggested that RGC_CA-like characters rarely exist in many states so RGC_CAs are truly rare genomics changes, although they are prone to homoplasy as (almost) any other phylogenetic character. The question is the homoplasy level which, in our opinion, is acceptable for RGC_CAs. We cannot guarantee that other sites in alignments have a homoplasy level low enough for efficient use of phylogenetic methods for long evolutionary distances. In this case heuristic attempts to shred long branches into shorter ones using as many species as possible might be important*.

In addition to the mixing of RNA helicase and of carbamoyl-phosphate synthetase in

Figure 1, there are some inconsistencies in this manuscript:

• In additional file 2, a lizard is present, but this is not indicated in the M&M.

*Authors' response: Corrected*.

• "To the resulting mixed C/KOGs, probable orthologs from three other eukaryotic genomes, namely, those of rice *Oryza sativa *(Os), moss *Physcomitrella patens *(Pp), chicken *Gallus gallus *(Gg), and red algae *Cyanidioschyzon merolae *(Cm) were added using COGNITOR." Replace three by four.

Authors' response: Corrected

• In the legend of Figure 4, "Estimates for three calibration points are shown." But I am unable to see them.

*Authors' response: We added an explanation: "Results obtained for all three calibration intervals (Figure *2*) were pooled together"*.

### Reviewer 3: Romain Derelle (Université Pierre et Marie Curie)

This reviewer provided no comments for publication.

## Supplementary Material

Additional file 1 **Time estimates using different programs, substitution models, sets of species and tree topologies**. Click here for file

Additional file 2 **The phylogenetic tree of vertebrates used for the analysis of approximate molecular clock properties of the RGC_CA approach**. Click here for file

Additional file 3 **A histogram of divergence time estimates for all employed methods**. Click here for file

Additional file 4 **Lengths of paths from LECA to terminal branches**. Click here for file

Additional file 5 **The eukaryotic phylogeny [the red alga *Cyanidioschyzon merolae *(Cm) included] adopted in this study**. Click here for file

Additional file 6 **A histogram of divergence time estimates for all employed methods obtained with the plant, insect and red algal calibration intervals**. Click here for file

Additional file 7 **Classification of 8 major prokaryotic groups (from the COG database) used as outgroups**. Click here for file
