# Supplementary material for: A late origin of the extant eukaryotic diversity: divergence time estimates using rare genomic changes
Source: Biol Direct. 2011 May 19;6:26. doi: 10.1186/1745-6150-6-26 (PMC3125394; doi:10.1186/1745-6150-6-26)
Supplement: Additional file 2 — The phylogenetic tree of vertebrates used for the analysis of approximate molecular clock properties of the RGC_CA approach. [file 1745-6150-6-26-S2.DOC]

Additional file 2. The phylogenetic tree of vertebrates used for the analysis of approximate molecular clock properties of the RGC_CA approach. The branch lengths are indicated in the RGC_CA numbers (A) and Dayhoff distances (multiplied by 10^3^) (B). Dayhoff distances were calculated using ungapped concatenated sequence alignments and the CODEML program.

(A)

Hs Mm Cf Md Oa Gg Ac Xt Dr

**human mouse dog opossum platypus chicken lizard toad fish**

Outgroup

At,Ce,Dm,

Sc,Sp,Pf,Ag

*62-101*

*95-113*

*125-138*

*260-300*

*161-191*

*312-330*

*330-*

*350*

**7**

**17**

**1**

**5**

**4**

**18**

**22**

**3**

**24**

**30**

**3**

**4**

**39**

**13**

**2**

*416-422*

**31**

**44**

(B)

Hs Mm Cf Md Oa Gg Ac Xt Dr

Outgroup

At,Ce,Dm,

Sc,Sp,Pf,Ag

*62-101*

*95-113*

*125-138*

*260-300*

*161-191*

*312-330*

*330-*

*350*

**8**

**26**

**4**

**12**

**59**

**141**

**128**

**4**

**118**

**113**

**3**

**18**

**97**

**43**

**38**

*416-422*

**147**

**178**

**human mouse dog opossum platypus chicken lizard toad fish**

Correlation between the cumulative branch lengths and midpoints of time estimates for RGC_CAs (A) and Dayhoff distances (multiplied by 10^-3^) (B). Dayhoff distances were calculated using ungapped concatenated sequence alignments and the CODEML program.

(A)


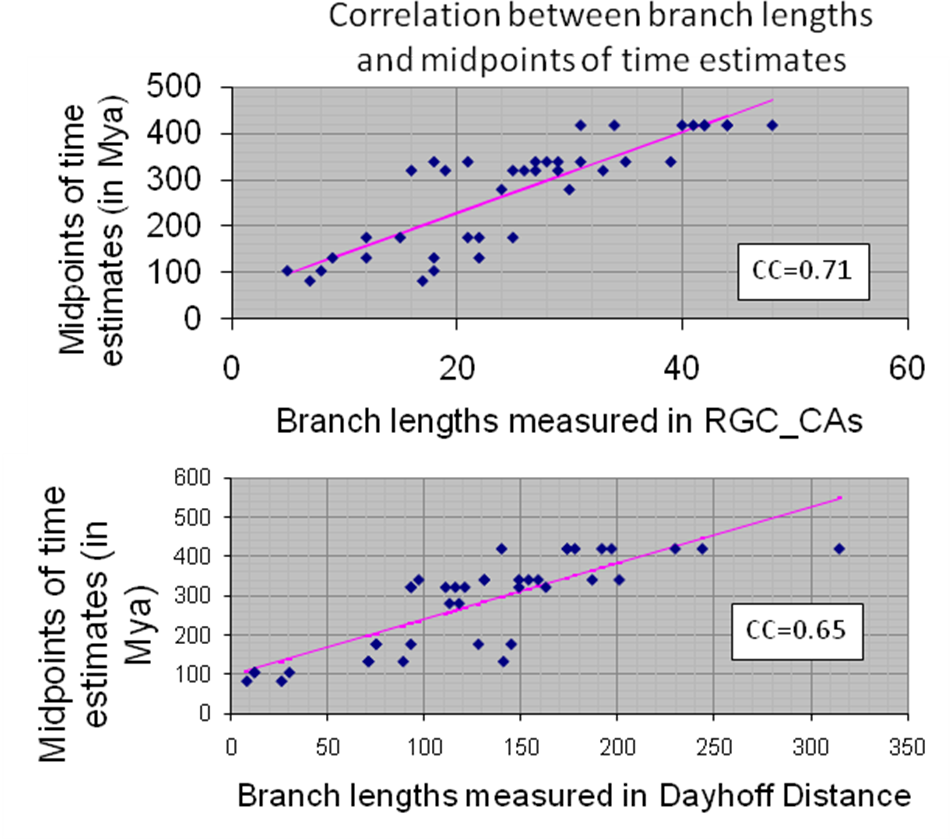


(B)
