# Supplementary material for: A late origin of the extant eukaryotic diversity: divergence time estimates using rare genomic changes
Source: Biol Direct. 2011 May 19;6:26. doi: 10.1186/1745-6150-6-26 (PMC3125394; doi:10.1186/1745-6150-6-26)

Additional file 3. A histogram of divergence time estimates for all employed methods (the data for red algae not included). Time estimates for maximum parsimony with the chicken calibration point (370 Mya) are shown in light blue.


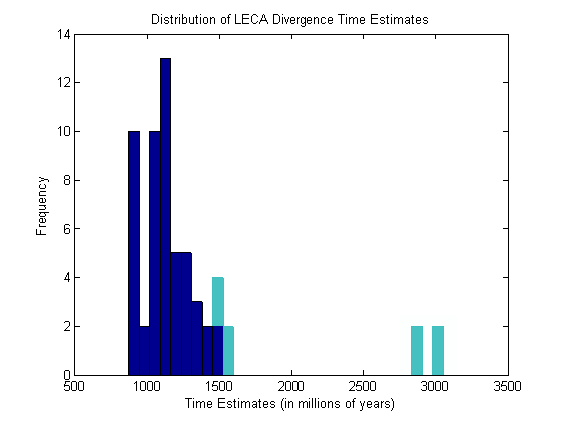


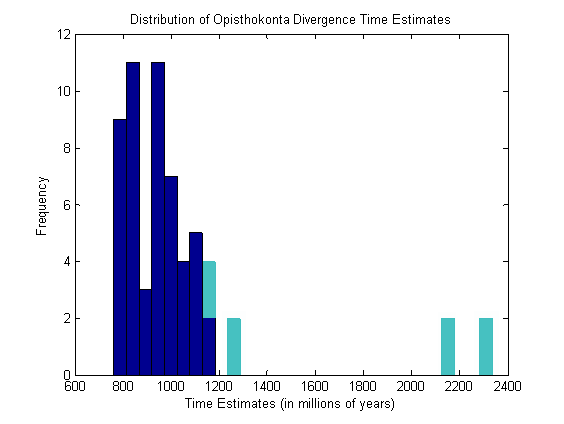


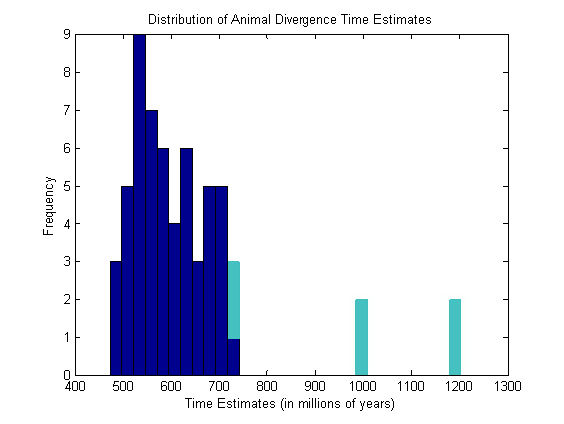


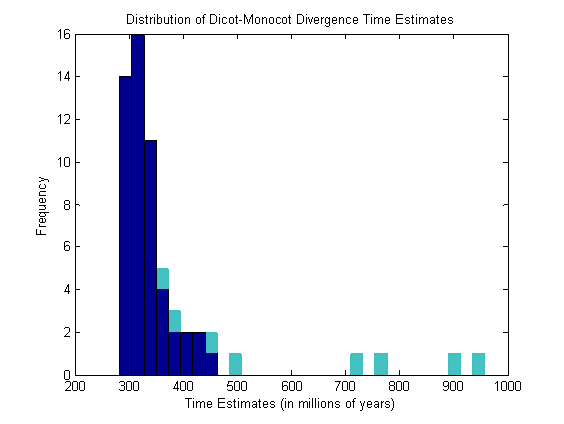

Supplement: Additional file 3 — A histogram of divergence time estimates for all employed methods. [file 1745-6150-6-26-S3.DOC]
