# Supplementary material for: A late origin of the extant eukaryotic diversity: divergence time estimates using rare genomic changes
Source: Biol Direct. 2011 May 19;6:26. doi: 10.1186/1745-6150-6-26 (PMC3125394; doi:10.1186/1745-6150-6-26)
Supplement: Additional file 4 — Lengths of paths from LECA to terminal branches. [file 1745-6150-6-26-S4.DOC]

Additional file 4. Lengths of paths from LECA to terminal branches (number of RGC_CAs).

|  | Pp | At | Os | Sc | Sp | Gg | Hs | Ag | Dm | Ce |
| --- | --- | --- | --- | --- | --- | --- | --- | --- | --- | --- |
|  |  |  |  |  |  |  |  |  |  |  |
| Min | 99 | 118 | 108 | 211 | 197 | 143 | 123 | 149 | 156 | 254 |
| Max | 99 | 118 | 108 | 211 | 197 | 152 | 132 | 157 | 164 | 237 |
|  |  |  |  |  |  |  |  |  |  |  |

Comparison of lengths of paths from LECA to terminal branches using the χ^2^ test (χ^2^ values and P values are shown).

|  | **At** | **Os** | **Sc** | **Sp** | **Gg** |  | **Hs** |  | **Ag** |  | **Dm** |  | **Ce** |  |
| --- | --- | --- | --- | --- | --- | --- | --- | --- | --- | --- | --- | --- | --- | --- |
| **Pp** | 1.66 | 0.39 | 40.46 | 32.45 | 8.00 | 11.19 | 2.59 | 4.71 | 13.14 | 10.08 | 16.06 | 12.74 | 11.96 | 56.68 |
|  | 0.20 | 0.53 | 0.00 | 0.00 | 0.00 | 0.00 | 0.11 | 0.03 | 0.00 | 0.00 | 0.00 | 0.00 | 0.00 | 0.00 |
| **At** |  | 0.44 | 26.29 | 19.81 | 2.39 | 4.28 | 0.10 | 0.78 | 5.53 | 3.60 | 7.50 | 5.27 | 4.76 | 39.89 |
|  |  | 0.51 | 0.00 | 0.00 | 0.12 | 0.04 | 0.75 | 0.38 | 0.02 | 0.06 | 0.01 | 0.02 | 0.03 | 0.00 |
| **Os** |  |  | 33.26 | 25.97 | 4.88 | 7.45 | 0.97 | 2.40 | 9.06 | 6.54 | 11.53 | 8.73 | 8.08 | 48.23 |
|  |  |  | 0.00 | 0.00 | 0.03 | 0.01 | 0.32 | 0.12 | 0.00 | 0.01 | 0.00 | 0.00 | 0.00 | 0.00 |
| **Sc** |  |  |  | 0.48 | 13.06 | 9.59 | 23.19 | 18.20 | 7.92 | 10.68 | 5.89 | 8.24 | 8.90 | 1.51 |
|  |  |  |  | 0.49 | 0.00 | 0.00 | 0.00 | 0.00 | 0.00 | 0.00 | 0.02 | 0.00 | 0.00 | 0.22 |
| **Sp** |  |  |  |  | 8.58 | 5.80 | 17.11 | 12.84 | 4.52 | 6.66 | 3.02 | 4.76 | 5.27 | 3.69 |
|  |  |  |  |  | 0.00 | 0.02 | 0.00 | 0.00 | 0.03 | 0.01 | 0.08 | 0.03 | 0.02 | 0.05 |
| **Gg** |  |  |  |  |  |  | 1.50 | 1.41 | 0.65 | 0.03 | 1.44 | 0.05 | 0.41 | 18.57 |
|  |  |  |  |  |  |  | 0.22 | 0.24 | 0.42 | 0.86 | 0.23 | 0.82 | 0.52 | 0.00 |
| **Hs** |  |  |  |  |  |  |  |  | 4.13 | 1.03 | 5.86 | 2.00 | 3.47 | 29.88 |
|  |  |  |  |  |  |  |  |  | 0.04 | 0.31 | 0.02 | 0.16 | 0.06 | 0.00 |
| **Ag** |  |  |  |  |  |  |  |  |  |  | 0.15 | 0.16 | 0.03 | 20.06 |
|  |  |  |  |  |  |  |  |  |  |  | 0.70 | 0.69 | 0.86 | 0.00 |
| **Dm** |  |  |  |  |  |  |  |  |  |  |  |  | 0.31 | 16.69 |
|  |  |  |  |  |  |  |  |  |  |  |  |  | 0.57 | 0.00 |
