# Supplementary material for: A late origin of the extant eukaryotic diversity: divergence time estimates using rare genomic changes
Source: Biol Direct. 2011 May 19;6:26. doi: 10.1186/1745-6150-6-26 (PMC3125394; doi:10.1186/1745-6150-6-26)

Additional file 5. The eukaryotic phylogeny [the red alga *Cyanidioschyzon merolae* (Cm) included] adopted in this study (the coelomate scenario, the ecdysozoa scenario is not shown). The numbers at the branches indicate the numbers of RGC_CAs which are used to measure the branch length. Tc, calibration time interval, Mya.


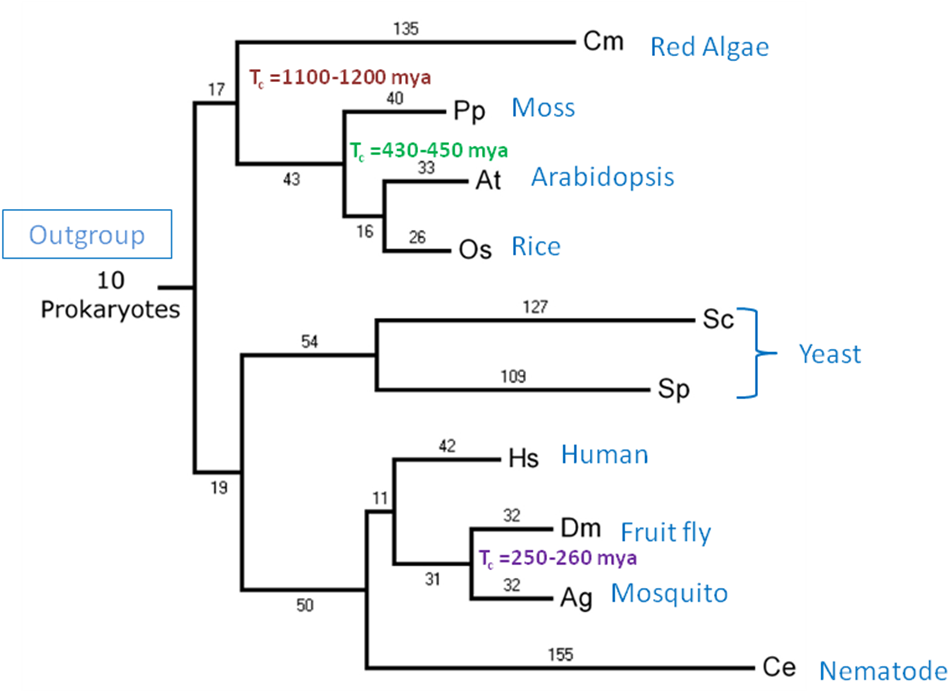

Supplement: Additional file 5 — The eukaryotic phylogeny [the red alga Cyanidioschyzon merolae (Cm) included] adopted in this study. [file 1745-6150-6-26-S5.DOC]
