# Supplementary material for: A late origin of the extant eukaryotic diversity: divergence time estimates using rare genomic changes
Source: Biol Direct. 2011 May 19;6:26. doi: 10.1186/1745-6150-6-26 (PMC3125394; doi:10.1186/1745-6150-6-26)
Supplement: Additional file 6 — A histogram of divergence time estimates for all employed methods obtained with the plant, insect and red algal calibration intervals. [file 1745-6150-6-26-S6.DOC]

Additional file 6. A histogram of divergence time estimates for all employed methods obtained with the plant, insect and red algal calibration intervals.
