# Supplementary material for: A late origin of the extant eukaryotic diversity: divergence time estimates using rare genomic changes
Source: Biol Direct. 2011 May 19;6:26. doi: 10.1186/1745-6150-6-26 (PMC3125394; doi:10.1186/1745-6150-6-26)
Supplement: Additional file 7 — Classification of 8 major prokaryotic groups (from the COG database) used as outgroups. [file 1745-6150-6-26-S7.DOC]

Additional file 7. Classification of 8 major prokaryotic groups (from the COG database) used as outgroups.

1) Afu,Hbs,Mac,Mth,Mja,Mka,Tac,Tvo,Pho,Pya,Sso,Ape (archaea)

2) Aae,Tma,Syn,Nos,Fnu,Dra (diverse bacteria)

3) Cgl,Mtu,Mle (actinobacteria)

4) Cac,Lla,Spy,Spn,Sau,Lin,Bsu,Bha,Uur,Mpu,Mpn,Mge (firmicutes)

5) Eco,Ype,Sty,Buc,Vch,Pae,Hin,Pmu,Xfa (gamma-proteobacteria)

6) Nme,Rso,Hpy,Cje (beta/epsilon-proteobacteria)

7) Atu,Sme,Bme,Mlo,Ccr,Rpr,Rco (alpha-proteobacteria)

8) Ctr,Cpn,Tpa,Bbu (Chlamydia-spirochetes)

**Species abbreviations:**

Afu *Archaeoglobus fulgidus*

Hbs *Halobacterium sp. NRC-1*

Mac *Methanosarcina acetivorans str.C2A*

Mth *Methanothermobacter thermautotrophicus*

Mja *Methanococcus jannaschii*

Mka *Methanopyrus kandleri AV19*

Tac *Thermoplasma acidophilum*

Tvo *Thermoplasma volcanium*

Pho *Pyrococcus horikoshii*

Pab *Pyrococcus abyssi*

Pya *Pyrobaculum aerophilum*

Sso *Sulfolobus solfataricus*

Ape *Aeropyrum pernix*

Aae *Aquifex aeolicus*

Tma *Thermotoga maritima*

Syn *Synechocystis*

Nos *Nostoc sp. PCC 7120*

Fnu *Fusobacterium nucleatum*

Dra *Deinococcus radiodurans*

Cgl *Corynebacterium glutamicum*

Mtu *Mycobacterium tuberculosis H37Rv*

MtC *Mycobacterium tuberculosis CDC1551*

Mle *Mycobacterium leprae*

Cac *Clostridium acetobutylicum*

Lla *Lactococcus lactis*

Spy *Streptococcus pyogenes M1 GAS*

Spn *Streptococcus pneumoniae TIGR4*

Sau *Staphylococcus aureus N315*

Lin *Listeria innocua*

Bsu *Bacillus subtilis*

Bha *Bacillus halodurans*

Eco *Escherichia coli K12*

Ype *Yersinia pestis*

Sty *Salmonella typhimurium LT2*

Buc *Buchnera sp. APS*

Vch *Vibrio cholerae*

Pae *Pseudomonas aeruginosa*

Hin *Haemophilus influenzae*

Pmu *Pasteurella multocida*

Xfa *Xylella fastidiosa 9a5c*

Nme *Neisseria meningitidis MC58*

Rso *Ralstonia solanacearum*

Hpy *Helicobacter pylori 26695*

Cje *Campylobacter jejuni*

Atu *Agrobacterium tumefaciens strain C58 (Cereon)*

Sme *Sinorhizobium meliloti*

Bme *Brucella melitensis*

Mlo *Mesorhizobium loti*

Ccr *Caulobacter vibrioides*

Rpr *Rickettsia prowazekii*

Rco *Rickettsia conorii*

Ctr *Chlamydia trachomatis*

Cpn *Chlamydophila pneumoniae CWL029*

Tpa *Treponema pallidum*

Bbu *Borrelia burgdorferi*

Uur *Ureaplasma urealyticum*

Mpu *Mycoplasma pulmonis*

Mpn *Mycoplasma pneumoniae*

Mge *Mycoplasma genitalium*
